# Epidemic Bronchocele

**Published:** 1901-10-26

**Authors:** 


### EPIDEMIC BRONCHOCELE.

Notwithstanding the knowledge which has of late years been gained in regard to the thyroid gland and the serious consequences which follow interference with its function, we remain in practically complete ignorance of the causes which lead to that enlargement of the thyroid which goes by the name of goitre. That this disease is so much more prevalent in some districts than in others as to be fairly describable as endemic in certain places is well known, but as to the cause of this endemicity we have to confess our ignorance. Endless causes have been suggested, each apparently upon good grounds so far as regards its own particular instance, but almost universally these explanations have failed to hold good in other cases, so that they have gained no general acceptance.

Here and there instances have been recorded in which the disease had seemed to take on an epidemic form, affecting considerable numbers of people almost simultaneously, and then, after a time, passing away. Such bronchocele epidemics have generally occurred in places where the disease has been reputed to be more or less endemic. Sometimes garrisons of young soldiers have been the sufferers, sometimes inmates of a school, sometimes people staying at some boarding house. Yet, although occurring in districts where the disease tends to be endemic, these epidemic outbreaks have not shown any tendency to spread beyond the institutions in which they have developed. Water supply has always been suspected as the cause, but whether the injurious element lies in some chemical or some vegetable impurity (if it lie in the water at all) seems quite unknown. Mr. Hutchinson[1] has lately reported the occurrence of an epidemic prevalence of bronchocele at a school at Witley, where he says, at one time, at least a third of the scholars were the subjects of enlargement of the thyroid gland. The epidemic began in a school which had been established in the same premises for many years, and without any known alteration in water supply, food, or other hygienic condition, and it subsided without any known influence to cause its decline. As in other like instances, the form of goitre was that known as parenchymatous or hyperplastic, consisting in a general enlargement or fulness of the whole gland. The facts would certainly suggest a drinking water origin, and it seems reasonable to suspect that the injurious material, whatever it may be, may be of vegetable origin although not necessarily of vegetable nature. In saying this, we have in our minds the phenomena of lead poisoning as seen in certain large "soft-water towns" in the North of England. In these towns lead poisoning sometimes occurs in what may properly be described as epidemics. Although the poison is of a purely mineral nature the poisoning breaks out in epidemics according to certain changes in the vegetable constituents of the soil on the great moorland gathering grounds whence the water flows; probably by way of their inducing in the water varying forms of acidity, by which the lead is oxydised and then brought into solution. Several instances, again, have been reported of late years of public water supplies—usually of pretty good character—suddenly becoming polluted by an enormous growth of diatoms or confervæ, and after a short time reverting again to their normal condition. Thus many of the facts of the case would be explicable on the hypothesis of a vegetable pollution of the water supply being at the root of the matter, although, as we have above suggested, this would not of necessity imply that the poison itself was of a vegetable nature. The whole matter is one of great interest, for in view of the known relation of the functions of the thyroid gland with many nutritive and developmental processes, all questions touching upon goitre have an importance which extends far beyond its merely æsthetic relations.

[1] Polyclinic, Oct.

